# Impaired autophagy in mouse embryonic fibroblasts null for Krüppel-like Factor 4 promotes DNA damage and increases apoptosis upon serum starvation

**DOI:** 10.1186/s12943-015-0373-6

**Published:** 2015-05-06

**Authors:** Changchang Liu, Elise P DeRoo, Catherine Stecyk, Margaret Wolsey, Mateusz Szuchnicki, Engda G Hagos

**Affiliations:** Department of Biology, Colgate University, 13 Oak Dr., Olin Hall 205A, Hamilton, NY 13346 USA; Program in Cellular and Molecular Medicine, Boston Childrens Hospital, Boston, MA 02115 USA; School of Medical School, Harvard University, Boston, MA 02115 USA; Cummings School of Veterinary Medicine, Tufts University, North Grafton, MA 01536 USA

**Keywords:** Krüppel-like Factor 4, Autophagy, Apoptosis, Mammalian target of rapamycin, DNA damage, Mouse embryonic fibroblasts, Autophagy related genes

## Abstract

**Background:**

Autophagy is a major cellular process by which cytoplasmic components such as damaged organelles and misfolded proteins are recycled. Although low levels of autophagy occur in cells under basal conditions, certain cellular stresses including nutrient depletion, DNA damage, and oxidative stress are known to robustly induce autophagy. Krüppel-like factor 4 (KLF4) is a zinc-finger transcription factor activated during oxidative stress to maintain genomic stability. Both autophagy and KLF4 play important roles in response to stress and function in tumor suppression.

**Methods:**

To explore the role of KLF4 on autophagy in mouse embryonic fibroblasts (MEFs), we compared wild-type with *Klf4* deficient cells. To determine the levels of autophagy, we starved MEFs for different times with Earle’s balanced salts solution (EBSS). Rapamycin was used to manipulate mTOR activity and autophagy. The percentage of cells with γ-H2AX foci, a marker for DNA damage, and punctate pattern of GFP-LC3 were counted by confocal microscopy. The effects of the drug treatments, *Klf4* overexpression, or *Klf4* transient silencing on autophagy were analyzed using Western blot. Trypan Blue assay and flow cytometry were used to study cell viability and apoptosis, respectively. qPCR was also used to assay basal and the effects of *Klf4* overexpression on *Atg7* expression levels.

**Results:**

Here our data suggested that *Klf4*^−/−^ MEFs exhibited impaired autophagy, which sensitized them to cell death under nutrient deprivation. Secondly, DNA damage in *Klf4*-null MEFs increased after treatment with EBSS and was correlated with increased apoptosis. Thirdly, we found that *Klf4*^−/−^ MEFs showed hyperactive mTOR activity. Furthermore, we demonstrated that rapamycin reduced the increased level of mTOR in *Klf4*^−/−^ MEFs, but did not restore the level of autophagy. Finally, re-expression of *Klf4* in *Klf4* deficient MEFs resulted in increased levels of LC3II, a marker for autophagy, and *Atg7* expression level when compared to GFP-control transfected *Klf4*^−/−^ MEFs.

**Conclusion:**

Taken together, our results strongly suggest that KLF4 plays a critical role in the regulation of autophagy and suppression of mTOR activity. In addition, we showed that rapamycin decreased the level of mTOR in *Klf4*^−/−^ MEFs, but did not restore autophagy. This suggests that KLF4 regulates autophagy through both mTOR-dependent and independent mechanisms. Furthermore, for the first time, our findings provide novel insights into the mechanism by which KLF4 perhaps prevents DNA damage and apoptosis through activation of autophagy.

**Electronic supplementary material:**

The online version of this article (doi:10.1186/s12943-015-0373-6) contains supplementary material, which is available to authorized users.

## Introduction

Krüppel-like factor 4 (KLF4) is a zinc-finger transcription factor with diverse regulatory functions in proliferation, differentiation, apoptosis, and development [1,2]. In many human cancers KLF4 is regarded as a tumor suppressor. For instance, the expression of *KLF4* is downregulated in bladder, lung, pancreatic, colorectal, gastric, esophageal, and prostate cancers [3–7]. Interestingly, in contrast to its role as a tumor suppressor, in many cancer types KLF4 may act as an oncogene in a context dependent manner, as it is overexpressed in primary breast ductal carcinoma and oral squamous cell carcinoma [8,9].

*In vitro*, mouse embryonic fibroblasts (MEFs) lacking *Klf4* have been shown to exhibit various abnormal cellular processes including increased rate of proliferation, oxidative stress-induced DNA damage and elevated levels of apoptosis [10,11]. Furthermore, *KLF4* expression is associated with conditions that initiate cell cycle arrest such as contact inhibition and serum deprivation [12]. KLF4 is thought to play an important role in regulating normal physiological processes by initiating cell cycle checkpoints as induction of KLF4 following DNA damage arrests cells at G1/S and G2/M checkpoints [13]. We have previously shown that a lack of *Klf4* in MEFs leads to increased genomic instability such as DNA double stranded breaks, aneuploidy, chromosome aberration, and centrosome amplification [10]. This genomic instability can be corrected by reintroduction of *Klf4* to *Klf4*^−/−^ MEFs [14]. More recently we have shown that KLF4 regulates the levels of reactive oxygen species (ROS) and prevents accumulation of DNA damage [11]. However, other possible mechanisms through which KLF4 maintains genomic stability await further investigation.

Autophagy is a conserved intracellular process that involves the degradation of cytoplasmic components inside lysosomes [15]. It is induced in response to stress and nutrient starvation to maintain cellular homeostasis by removing misfolded proteins and damaged organelles so that the cell can recycle its components [16]. Both basal level and starvation-induced autophagy maintain cellular homeostasis and perform important housekeeping functions in a variety of physiological processes and pathological conditions including cancer [17]. Previous studies have shown that autophagy suppresses tumorigenesis. For example, monoallelic deletions of *beclin1,* a gene important for autophagy, lead to many types of cancer including ovarian, breast, and prostate cancer [18]. Furthermore, deletion of genes that result in autophagy deficiency in mice also accelerates tumor progression [19,20]. One mechanism by which autophagy is thought to suppress tumor development is by clearing damaged cellular components and mitigating cellular stress [21]. For instance, the autophagy defective *Beclin*^*+/−*^ mouse kidney epithelial cells showed increased DNA damage, centrosome amplification, and chromosomal abnormality, a hallmark of genomic instability [22]. However, the role autophagy plays in cancer development remains controversial. While autophagy plays a role in maintaining normal homeostasis in healthy tissues, studies have also reported that autophagy allows for the survival of cancer cells with defective apoptosis [17].

One crucial step of autophagy is the formation of autophagosomes, which engulf cytoplasmic components and transfer them to the lysosome for degradation. The formation of autophagosome involves the recruitment of lipidated Microtubule-associated protein 1 light chain 3 (LC3, homologue of yeast ATG8). LC3 is first translated as pro-LC3, which is later cleaved at the C-terminal to produce LC3I by ATG4B [23,24]. During starvation when autophagy is induced, cytosolic LC3I is conjugated to phosphatidylethanolamine (PE) to produce LC3II via enzymatic reactions involving an E1 activation enzyme ATG7 [23,25]. PE-conjugated LC3II then binds to the membranes of the elongating autophagosome and might participate in selective intake of target molecules [24,26]. Localization of LC3II to the autophagosome exhibits punctate pattern by microscopy [24]. Thus, conversion to LC3II and its punctate pattern have been used as good indicators for autophagic activity [24].

Autophagy can be regulated by mammalian target of rapamycin (mTOR). The mTOR complex is sensitive to the intracellular abundance of growth factors and nutrients. When nutrients are abundant, energy is high, and cellular stress is low, mTOR is active and ensures that anabolic pathways favor cell growth [27]. During normal physiological conditions, mTOR inhibits autophagy, and stimulates cell growth by phosphorylating p70S6K [28]. However, under nutrient deprivation and cellular stress, mTOR is inhibited and autophagy is induced [16,29]. Previous studies have shown that mTOR hyperactivity is implicated in many cancers. For example, mTOR is overexpressed in colorectal cancers, and several carcinomas are found to contain a single amino acid mutation that leads to mTOR constitutive activation [30,31]. Further understanding of the mTOR pathway, as well as its negative regulators, thus may provide important insights into their role in carcinogenesis.

Despite the growing evidence supporting KLF4’s role in preventing tumorigenesis, the exact mechanism by which it exerts such functions remains unclear. In the present study, we sought to investigate whether KLF4 played a role in autophagy regulation and to identify its molecular targets. We propose here that primary MEFs lacking *Klf4* exhibited impaired autophagy during starvation, which sensitized the cells to apoptosis. We attributed the cause of autophagy impairment partially to mTOR hyperactivity and showed KLF4 as a negative regulator of mTOR. Results from this work can further elucidate the mechanism by which KLF4 maintains genomic stability and limits carcinogenesis through activation of autophagy.

## Results

### MEFs lacking *Klf4* exhibited impaired autophagy during starvation

**Figure 1:**
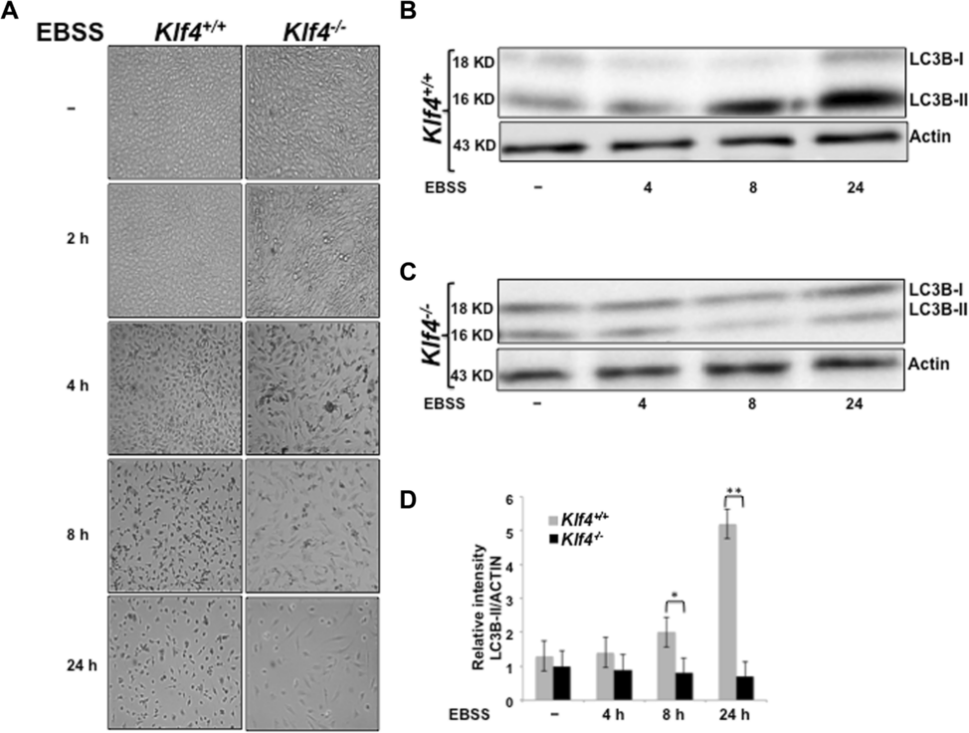
MEFs null for *Klf4* exhibit lower levels of autophagy compared to the wild-type during starvation. **(A)** Morphology of *Klf4* ^+/+^ and *Klf4* ^−/−^ MEFs with time course EBSS treatment. *Klf4* ^+/+^ and *Klf4* ^−/−^ MEFs were treated with EBSS for the durations indicated and photographed with an Olympus IX51 inverted microscope. Cell lysates from *Klf4* ^+/+^ **(B)** and *Klf4* ^−/−^ **(C)** MEFs were analyzed using Western blot. Antibodies against LC3B were used to indicate autophagy levels and β-Actin was used as a loading control. **(D)** Densitometry analysis of LC3B bands shown in **(B)** and **(C)**. The intensities of LC3BII bands were normalized to that of β-actin using Gel Imager program. Error bars represent standard error. The experiments were repeated 3 times. * indicates p < 0.05, ** indicates p < 0.01.

In order to explore their response to starvation conditions, MEFs were treated with Earle’s Balanced Salt Solution (EBSS) for various durations of time. Cell morphology was assessed by microscope in *Klf4*^+/+^and *Klf4*^−/−^ MEFs treated with either full-media or EBSS. As shown by the relative number of cells in Figure 1A, *Klf4*^−/−^ MEFs were more sensitive to the effects of starvation in time-dependent manner compared with wild-type MEFs. As a control, *Klf4*^+/+^and *Klf4*^−/−^ MEFs cultured in full media showed normal increase in confluency after 24 hrs (Additional file 1 Figure S1).

We next examined the basis of this increased sensitivity to starvation in *Klf4*^−/−^ MEFs. Previous reports have shown that autophagy is induced in response to nutrient starvation [16]. Consistent with those reports, Western blot analysis of the autophagy marker, LC3BII, showed that while *Klf4*^+/+^ MEFs significantly exhibited higher levels of autophagy after 8 hrs of treatment with EBSS, starvation failed to induce similar increases of autophagy in *Klf4*^−/−^ MEFs (Figures 1B—D). Additionally in the absence of *Klf4*, *Atg7*, a gene important for autophagy, exhibited reduced induction compared to that of the wild-type during starvation (data not shown). Our data thus strongly suggested that KLF4 mediates the autophagy process.

### Impaired autophagy sensitizes *Klf4*^*−/−*^ MEFs to apoptosis

**Figure 2:**
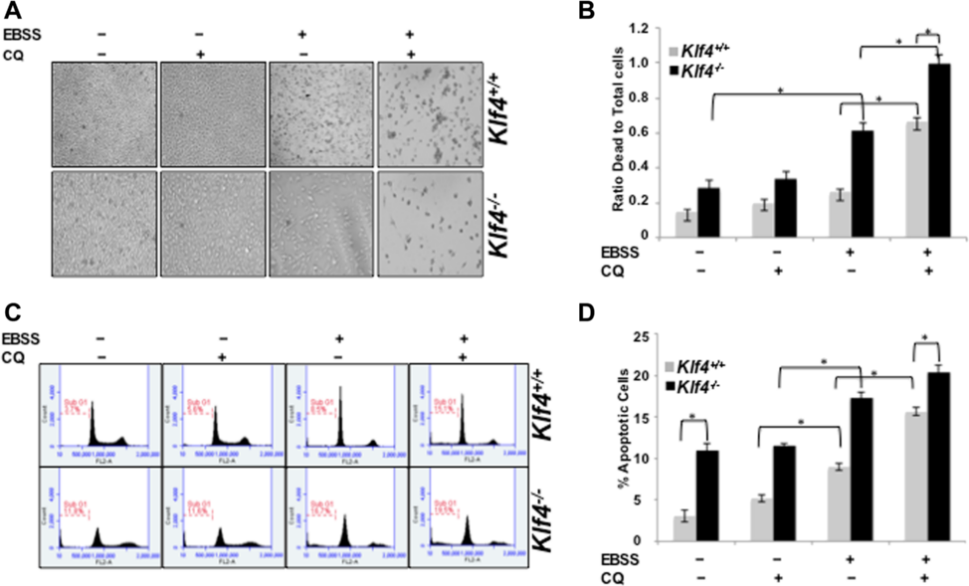
Impairment of autophagy in *Klf4* ^*−/−*^ MEFs leads to increased apoptotic cell death. **(A)** Morphology of *Klf4* ^+/+^ and *Klf4* ^−/−^ MEFs treated with full media, 10 μM CQ, EBSS, or both CQ and EBSS for 8 hrs. **(B)** MEFs were collected after respective treatments and subjected to Trypan Blue assay. The percentage of dead cells, as indicated by Trypan Blue staining, was quantified using a hemocytometer under bright field microscopy. Error bars represent standard deviation. The experiments were repeated 3 times. * indicates p < 0.05. **(C)** *Klf4* ^+/+^ and *Klf4* ^−/−^ MEFs were treated with full media, 10 μM CQ, EBSS, or both CQ and EBSS for 8 hrs before the cells were analyzed using flow cytometry. Representative results from three independent experiments were shown. Sub-G1 population was identified for quantification of cell survival. **(D)** Sub-G1 population from the three independent experiments represented in **(C)** was averaged and plotted. Error bars represent standard error. * indicates p < 0.05.

We next attempted to study the relative effects on cell viability in *Klf4*^+/+^ and *Klf4*^−/−^ MEFs caused by impaired autophagy using chloroquine diphosphate (CQ), which inhibited the completion of autophagy by blocking the maturation of the autophagosome and neutralizing cellular pH [32]. Relative cell number and Trypan Blue assay showed a significant increase in the percentage of dead cells in *Klf4*^−/−^ MEFs starved with EBSS (Figures 2A—B). Both *Klf4*^+/+^ and *Klf4*^−/−^ MEFs starved and co-treated with CQ showed increased percentage of dead cells (Figure 2B). The increased cell death was not likely to be caused by CQ toxicity as CQ treatment alone had little effect on cell viability (Figure 2B). The increased cell death in both *Klf4*^+/+^ and *Klf4*^−/−^ MEFs might be caused by apoptosis, necrosis, or autophagy-induced cell death. To determine whether inhibition of autophagy led to increased cell death by sensitizing cells to apoptosis, we performed flow cytometry. Consistent with our previous finding [10], we observed a significantly higher sub-G1 population of cells in *Klf4*^−/−^ MEFs compared to the wild-type (Figures 2C—D). During starvation, inhibition of autophagy increased the sub-G1 population from 8.5% to 15.1% in wild-type MEFs as compared to starvation only while the apoptotic population increased from 16.7% to 19.5% in *Klf4*^−/−^ MEFs (Figure 2C). Our results thus suggested that inhibition of autophagy increased apoptosis and that *Klf4*^−/−^ MEFs may be more sensitive to starvation due to impaired autophagy. However, the slight increase of apoptosis in *Klf4*^−/−^ MEFs with CQ and EBSS combined treatment does not fully explain their higher rate of cell death (Figure 2B&D). This suggested that *Klf4*^−/−^ MEFs might experience apoptosis-independent cell death.

### Impaired autophagy in *Klf4*^*−/−*^ MEFs results in increased DNA damage

**Figure 3:**
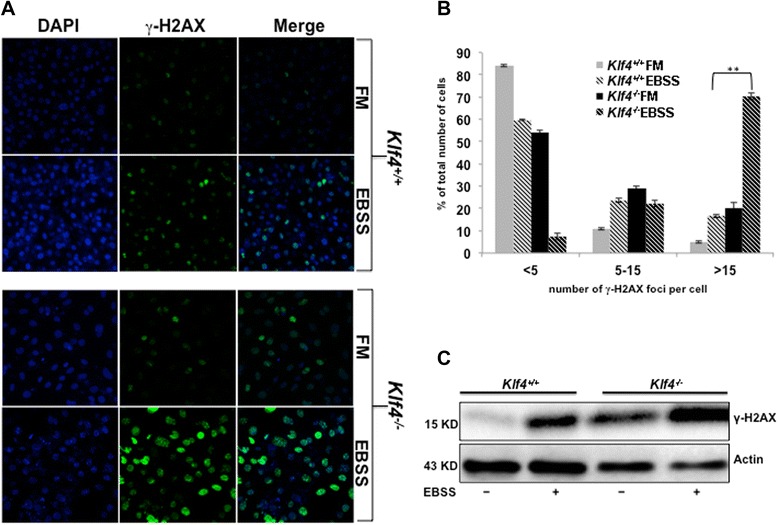
Impaired autophagy in *Klf4* ^*−/−*^ MEFs results in increased DNA damage. *Klf4* ^+/+^ and *Klf4* ^−/−^ MEFs were treated with full-media or EBSS for 8 hrs. **(A)** Immunostaining against γ-H2AX (green), a DNA damage marker, was performed to assess the levels of DNA damage in *Klf4* ^+/+^ and *Klf4* ^−/−^ MEFs. DAPI (blue) was used to visualize the nuclei. **(B)** The percentage of *Klf4* ^+/+^ and *Klf4* ^−/−^ MEFs cultured in full-media or EBSS with less than 5, 5—15, or more than 15 γ-H2AX foci was quantified. At least 300 MEFs were counted for each condition. * indicates p < 0.05, ** indicates p < 0.01. **(C)** Western blot analysis was performed to assay the levels of γ-H2AX. The experiments were repeated 3 times.

It has been previously established that extensive DNA damage can lead to apoptosis, and autophagy is an important mechanism in maintaining genomic stability [22,33,34]. We thus hypothesized that impaired autophagy in *Klf4*^−/−^ MEFs led to the accumulation of DNA damage and sensitized the cells to apoptosis. In order to test our hypothesis, we cultured *Klf4*^+/+^ and *Klf4*^−/−^ MEFs in full-media or starved them with EBSS. After 8 hours of EBSS treatment, the cells were immunostained with an antibody against S139-phophorylated H2AX (γ-H2AX), a DNA damage marker. Consistent with our previous findings at the basal level [10,14], *Klf4*^−/−^ MEFs exhibited higher levels of γ-H2AX foci compared to the wild-type (Figure 3). When challenged with starvation, *Klf4*^+/+^ MEFs showed a moderate increase in γ-H2AX foci (Figures 3A&B). However, more *Klf4*^−/−^ MEFs showed more severe levels of DNA damage (Figures 3A&B). Quantification of γ-H2AX foci showed that most of *Klf4*^+/+^ MEFs treated with EBSS contained 5 foci or fewer per cell and a small fraction had greater than 15 foci per cell. However, the majority of *Klf4*^−/−^ MEFs contained more than 15 foci per cell (Figure 3B). Furthermore, Western blot analysis of γ-H2AX also showed similar results. As shown in Figure 3C, the levels of γ-H2AX in EBSS treated *Klf4*^−/−^ MEFs is much higher as compared with wild-type cells (Figure 3C). Taken together, these results supported our hypothesis that defective autophagy in *Klf4*^−/−^ MEFs led to accumulation of DNA damage.

### MEFs lacking *Klf4* exhibit suppressed autophagy level due to mTOR hyperactivity

**Figure 4:**
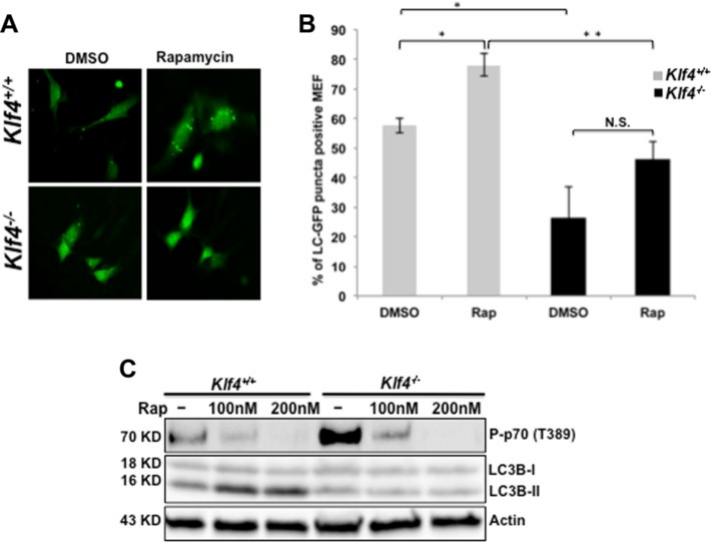
Rapamycin induced autophagy in *Klf4* ^+/+^ but not *Klf4* ^−/−^ MEFs. **(A)** *Klf4* ^+/+^ and *Klf4* ^−/−^ MEFs were seeded to 50-70% confluence and transfected with GFP-LC3. Five hours post-transfection, MEFs were treated with 100 nM rapamycin or DMSO for 24 hrs. **(B)** The percentage of cells in **(A)** positive for GFP-LC3B puncta was quantified. Error bars represent standard error. The experiments were repeated 3 times. * indicates p < 0.05, ** indicates p < 0.01. No significant difference in the levels of LC3II was observed in *Klf4* ^−/−^ MEFs treated with either DMSO or rapamycin (p = 0.249). **(C)** *Klf4* ^+/+^ and *Klf4* ^−/−^ MEFs were treated with rapamycin of indicated dosages or DMSO for 24 hrs. Cell lysates were analyzed by Western blot using antibodies against phospho-p70S6K and LC3B. β-Actin was used as a loading control. The experiments were repeated 3 times.

A previous study of vascular smooth muscle cells has shown that transient knockdown of *Klf4* led to increased levels of mTOR downstream targets [35]. Consequently we investigated whether the impaired autophagy in *Klf4*^−/−^ MEFs could be due to mTOR hyperactivity. To test our hypothesis, we first transfected *Klf4*^+/+^ and *Klf4*^−/−^ MEFs with GFP-LC3B plasmid and treated both MEFs with rapamycin, a known inhibitor of mTOR. The induction of autophagy correlates with the conversion of diffused LC3I in the cytoplasm to LC3II which localized to the autophagosome and results in punctate pattern [24]. Thus the levels of autophagy were visualized under a fluorescence microscope and quantified by the percentage of GFP-LC3B puncta. Consistent with Figures 1B—C, *Klf4*^−/−^ MEFs displayed lower basal levels of autophagy compared to *Klf4*^+/+^ MEFs (Figures 4A—B). While rapamycin increased the levels of autophagy in *Klf4*^+/+^ MEFs, no significant increase of LC3 puncta was observed *Klf4*^−/−^ MEFs (p = 0.249). Thus rapamycin was unable to restore the reduced levels of autophagy in MEFs lacking *Klf4* (Figures 4A—B). The molecular effect of rapamycin treatment was also analyzed by Western blot for assay of mTOR activity. At the basal level, phosphorylated p70S6K increased in *Klf4*^−/−^ MEFs compared to the wild-type, suggesting that the mTOR pathway in *Klf4*^−/−^ MEFs was hyperactive (Figure 4C). The elevated level of phosphorylated p70S6K was effectively abolished in response to rapamycin in both cell lines (Figure 4C). Interestingly, suppression of the hyperactive mTOR pathway by rapamycin restored the levels of autophagy in wild-type but not in *Klf4-*null MEFs (Figure 4C).

### Knockdown of *Klf4* reduces autophagy levels and makes MEFs resistant to the effects of rapamycin

**Figure 5:**
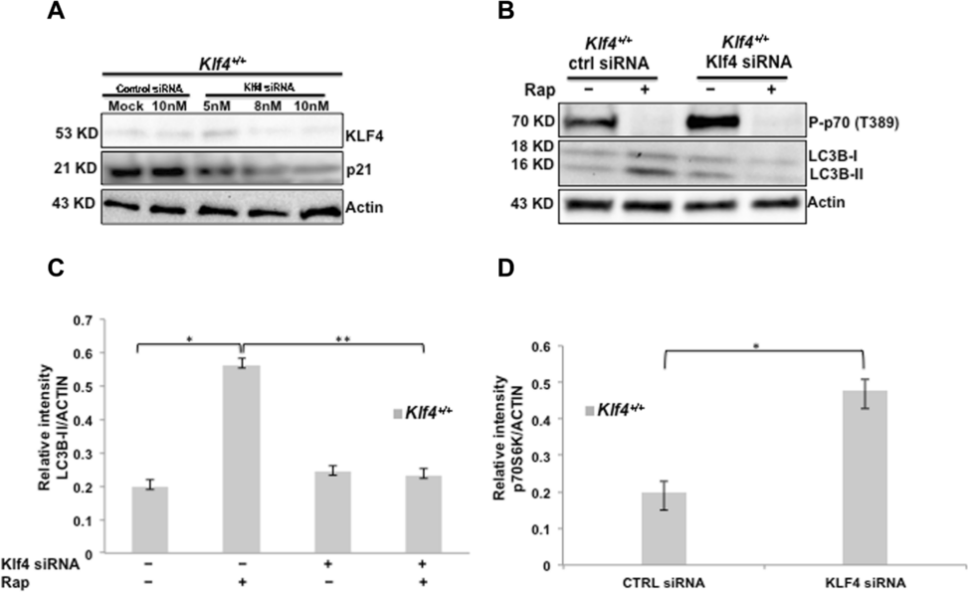
*Klf4* knockdown leads to decreased autophagy levels and elevated mTOR activity. **(A)** *Klf4* expression was inhibited by treating *Klf4* ^+/+^ MEFs with indicated concentrations of siRNA. Proteins were extracted from the cells 24 hrs after siRNA treatment. The levels of KLF4 and p21, a downstream target of KLF4, were assessed using Western blot. **(B)** *Klf4* ^+/+^ MEFs were treated with control siRNA or *Klf4* siRNA complex. Five hours post transfection, transfection media was replaced with full-media that contained 100 nM rapamycin or DMSO as a control. Proteins were extracted 24 hrs after rapamycin treatment and Western blot analysis was performed to assay phospho-p70S6K and LC3 levels. **(C)** Densitometry analysis of LC3B bands shown in **(B)**. The intensities of LC3BII bands were normalized to that of β-actin using Gel Imager program (Bio-Rad Laboratories, Hercules, CA). Error bars represent standard error. The experiments were repeated 3 times. * indicates p < 0.05, ** indicates p < 0.01. **(D)** Densitometry analysis of phospho-p70S6K bands shown in **(B)**. The intensities of phospho-p70S6K bands were normalized to that of β-Actin using Gel Imager program. Error bars represent standard error. The experiments were repeated 3 times. * indicates p < 0.05.

In order to examine whether KLF4 was necessary for autophagy induction, we attempted to transiently silence *Klf4* expression using siRNA against *Klf4* in wild-type MEFs. Figure 5A confirmed the successful knockdown of *Klf4* and the downregulation of its downstream target p21. Supporting our earlier observation in *Klf4-*null cells, knockdown of *Klf4* in wild-type MEFs significantly reduced autophagy level (Figures 5B&C). More importantly, when *Klf4* expression was abolished, rapamycin failed to induce autophagy even though it effectively suppressed the mTOR pathway, as indicated by the reduced levels of phospho-p70S6K (Figures 5B—D).

### *Klf4* re-expression in *Klf4*-null MEFs increases autophagy and decreases mTOR activity

**Figure 6:**
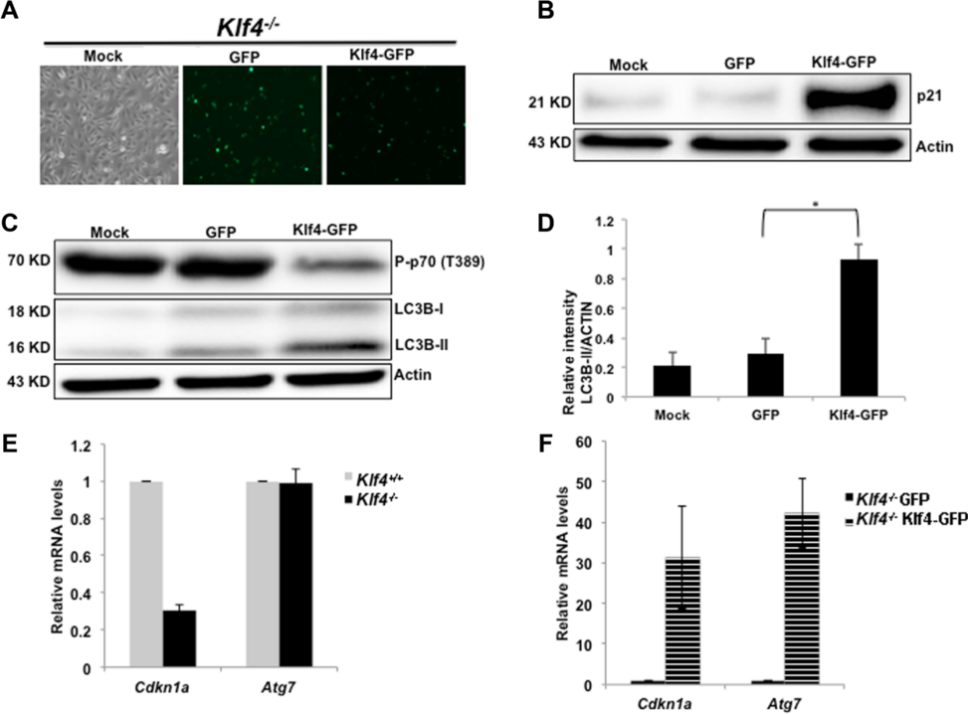
Overexpression of *Klf4* in *Klf4-null* MEFs restores autophagy levels. *Klf4* ^−/−^ MEFs were transfected with mock media, GFP, or Klf4-GFP plasmids. **(A)** GFP signal was visualized under an Olympus IX51 microscope. Proteins were extracted 24 hrs after transfection. The levels of p21 **(B)** phospho-p70S6K and LC3 **(C)** were assayed with Western blot analysis. **(D)** Densitometry analysis of LC3B bands shown in **(C)**. The intensities of LC3BII bands were normalized to that of β-actin using Gel Imager program. Error bars represent standard error. The experiments were repeated 3 times. * indicates p < 0.05. **(E)** RT-qPCR was performed to assess the expression of *Cdkn1a* as a positive control and *Atg7* at the basal level. The level of *Cdkn1a* or *Atg7* was normalized to *β-Actin*. The normalized expression level of *Cdkn1a* and *Atg7* was shown as relative fold change of the gene expression level in wild-type. **(F)** RT-qPCR was performed to assess the expression of *Cdkn1a* as a positive control and *Atg7* in *Klf4* ^−/−^ MEFs transfected with *Klf4*. The level of *Cdkn1a* or *Atg7* was normalized to *β-Actin*. The normalized expression level of *Cdkn1a* and *Atg7* was represented as relative fold change of the gene expression level in GFP alone or *Klf4* ^−/−^ MEFs transfected with Klf4-GFP. The experiment was done twice each in triplicate.

To examine whether KLF4 was sufficient for autophagy induction, we reintroduced *Klf4* to *Klf4*^−/−^ MEFs. Re-expression of *Klf4* in *Klf4*^−/−^ MEFs was confirmed via fluorescence microscopy by examining the green fluorescence protein (GFP) signal conjugated to *Klf4* and via Western blot by assessing the level of p21 (Figures 6A—B). Reintroduction of *Klf4* suppressed the downstream mTOR target, phospho-p70S6K, and induced LC3I to LC3II conversion, whereas mock treatment and GFP expression had a minimal effect on the level of phospho-p70S6K and autophagy (Figures 6C—D). In order to investigate the mechanism by which KLF4 mediates the conversion of LC3I to LC3II, we assayed the expression level of *Atg7*, which acts as an E1 enzyme to mediate the conversion from LC3I to LC3II [26]. Under normal conditions, no difference in *Atg7* expression was observed between *Klf4*^+/+^ and *Klf4*^−/−^ MEFs possibly due to minimal induction of autophagy when nutrients were optimal (Figure 6E). However, when KLF4 was reintroduced to *Klf4*^−/−^ MEFs, the expression of *Atg7* was upregulated, similar to that of the known KLF4 downstream transcription target, *Cdkn1a* which encodes a cyclin-dependent kinase inhibitor, p21. (Figure 6 F). Our data thus suggested that KLF4 might be involved in the autophagy process by regulating *Atg7* expression level.

## Discussion

It has been well-established that KLF4 acts as a tumor suppressor in many types of cancers [3–5,7,36]. One way KLF4 exerts its anti-tumorigenic function is by initiating cell cycle checkpoints during DNA damage and mediating p53 transactivation of p21 [13,37]. In addition to its role in inducing cell cycle arrest, we have also shown that KLF4 maintains genomic stability [10,14]. MEFs lacking *Klf4* have higher levels of genomic instability including DNA double stranded breaks, centrosome amplification as well as aneuploidy [10]. Restoration of KLF4 to *Klf4*^−/−^ MEFs significantly reduces DNA damage and aneuploidy [14]. More recently we have demonstrated that KLF4 reduces genomic instability by regulating antioxidant genes and reducing reactive oxygen species (ROS) [11].

Autophagy is a process by which long-lived proteins and organelles in the cytoplasm are degraded and recycled for cellular use. It can be triggered during nutrient deprivation and stress, but also plays an important role against the progression of some human diseases including cancer, infections, and neurodegenerative diseases [38]. A growing body of evidence shows connections between autophagy and genomic instability [17–22,33]. Autophagy removes misfolded proteins and damaged organelles to prevent cells from inducing the production of ROS [39,40]. As a result, cells deficient for genes required for autophagy exhibit higher genomic instability [33,40]. Furthermore, previous studies have shown that cells with defective autophagy due to hyperactive mTOR activity exhibited increased sensitivity to DNA damage agents [41].

We propose here that MEFs deficient in *Klf4* expression exhibit impaired autophagy (Figure 1). Moreover, MEFs lacking *Klf4* exhibit higher levels of DNA damage upon starvation as compared to wild-type MEFs (Figure 3). Our results are in accord with previous findings that impaired autophagy predisposes cells to genomic instability during metabolic stress [33,41]. We have previously proposed that KLF4 reduces oxidative DNA damage and reactive oxygen species by regulation of antioxidant genes [11]. In addition to our previously proposed mechanism, genomic instability in *Klf4*-null MEFs might also be attributed to impaired autophagy. Previous reports indicated that when normal cells are challenged with metabolic stress, autophagy is activated and damaged intracellular components are removed via lysosomes, alleviating stress [42]. Defective autophagy could lead to accumulation of malfunctioning mitochondria, which often causes increased production of ROS [42]. Thus the impairment of autophagy in *Klf4*^−/−^ MEFs may result in failure to mitigate metabolic stress and therefore could lead to accumulation of genomic instability. Our current study suggests that in addition to the initiation of cell cycle checkpoints, KLF4 might maintain genomic stability through the induction of autophagy, and that the regulation of autophagy may be a novel mechanism by which KLF4 prevents tumorigenesis.

We have previously reported that MEFs lacking *Klf4* experience higher rates of apoptosis compared to wild-type cells [10]. Consistent with our previous finding, we showed here that *Klf4*^−/−^ MEFs are more prone to apoptosis under starvation by EBSS (Figure 2). Our data thus strongly argues for a prosurvival role of autophagy under cellular stress. While autophagy helps wild-type cells to recycle nutrients and survive nutrient deprivation, *Klf4*^−/−^ MEFs could be more sensitive to starvation because of impaired autophagy and thus they are unable to optimize cellular nutrients. This is in accord with previous findings which report that inhibition of autophagy in MCF cells and *Tsc*^*−/−*^ MEFs increases apoptosis [43,44].

**Figure 7:**
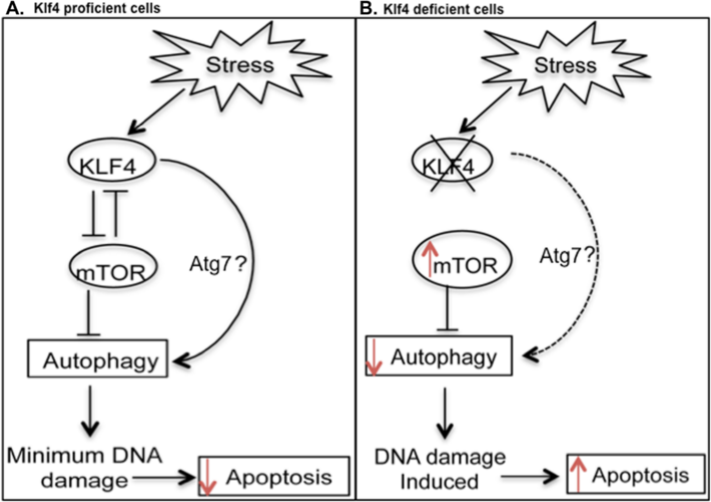
Diagraphic illustration of involvement of Klf4 in autophagy to prevent DNA damage induced apoptosis. This illustration outlines the regulation of autophagy in *Klf4* proficient and deficient cells during stress and places our results in the context of known and potential relationships between KLF4, mTOR, autophagy, DNA damage and apoptosis. Based on the present study and as previously shown in [35], in wild-type MEFs, KLF4 is induced under stress and might induce autophagy in mTOR dependent or independent manners. Autophagy then removes damaged cellular components and prevents DNA damage as well as apoptosis **(A)**. On the other hand, in MEFs lacking *Klf4*, autophagy induction is impaired due to mTOR hyperactivity and other unknown mechanisms. As a result, under starvation stress impaired autophagy promotes DNA damage and leads to increased apoptosis **(B)**. KLF4 might induce autophagy by negatively regulating mTOR. However, KLF4 may also regulate autophagy in an mTOR-independent mechanism, possibly involving regulations of autophagy-related genes such as Atg7.

Next, we attempted to investigate the molecular mechanism by which KLF4 participates in autophagy induction. mTOR negatively regulates the induction of autophagy by responding to nutrient as well as growth factor deprivation [29]. A study in vascular smooth muscle cells found that overexpression of *Klf4* reduced mTOR activity while transient silencing of *Klf4* led to increased mTOR activity [35]. Furthermore, knockdown of elF4GI, a downstream target of mTOR, led to increased levels of KLF4 and induced autophagy [45]. We thus hypothesized that reduced autophagy levels in *Klf4*^−/−^ MEFs could be due to mTOR hyperactivity. Supporting the findings in vascular smooth muscle cells, we report that MEFs lacking *Klf4* showed higher basal levels of mTOR activity (Figure 3C). Transient knockdown of *Klf4* led to elevated mTOR activity while restoration of *Klf4* into *Klf4*^−/−^ MEFs reduced mTOR activity (Figures 4—5). These findings thus suggest that KLF4 negatively regulates mTOR in MEFs (Figure 7).

One interesting observation of our study is that upon addition of rapamycin to wild-type and *Klf4-*null MEFs the mTOR pathway is highly downregulated. However, pharmacological induction of autophagy with rapamycin increases levels of autophagy only in *Klf4* wild-type, but not *Klf4-*null MEFs (Figures 4C&5B). This impairment of autophagy can be effectively rescued by transient expression of *Klf4* in the *Klf4-*null MEFs. This suggests that mTOR hyperactivity alone is not the only cause of the impaired autophagy in *Klf4*^−/−^ MEFs. One possibility is that KLF4 regulates autophagy via mTOR independent mechanisms potentially through autophagy-related genes (Atgs). We thus attempted to examine the expression of *Atg7*, a gene important for autophagosome expansion, and its knockdown prevented the conversion from LC3I to LC3II [44]. Our data supported this hypothesis, as restoration of KLF4 into *Klf4*^−/−^ MEFs led to upregulation of *Atg7* expression (Figure 6 F). This suggested that during starvation, KLF4 might mediate the transcription of *Atg7* to induce autophagy. However, whether KLF4 regulates *Atg7* via direct or indirect interaction awaits further investigation. Additionally our data did not eliminate the possibility that KLF4 might induce autophagy by regulating other autophagy-related genes. Our previous microarray data showed that *Klf4*^−/−^ MEFs exhibited enriched expression of autophagy related gene 3 (*Atg3*) [46]. However, we were not able to validate the microarray data via RT-PCR, as no difference was observed between *Klf4*^+/+^ and *Klf4*^−/−^ MEFs at the mRNA level (data not shown). Therefore, future studies remain necessary to understand the possible downstream targets through which KLF4 regulates autophagy.

## Conclusion

In summary, the present study suggested that MEFs lacking *Klf4* exhibited impaired autophagy, which resulted in their higher sensitivity to nutrient deprivation and increased apoptosis. This impaired autophagy in *Klf4*^−/−^ MEFs could be explained partially by mTOR hyperactivity, as KLF4 negatively regulates mTOR. However, other means by which KLF4 regulates autophagy independently of mTOR may exist and future studies should be devoted to shed light on these molecular mechanisms. The high level of DNA damage observed in *Klf4*^−/−^ MEFs during starvation could indicate that KLF4 is required to maintain genomic stability during metabolic and oxidative stress. Collectively, these findings provide new insights into the mechanism by which KLF4 maintains genomic stability and protects the cells from apoptosis (Figure 7).

## Materials and Methods

### Cell Culture, Reagents and Drug Treatments

Mice heterozygous for the Klf4 alleles (Klf4+/−) on a C57BL/6 background were crossbred [47]. MEFs that are wild-type (*Klf4*^*+/+*^), heterozygous (*Klf4*^*+/−*^), or null (*Klf4*^*−/−*^) for *Klf4* were derived from day 13.5 embryos. MEFs were subsequently cultured in Dulbecco’s modified Eagle’s medium (DMEM) supplemented with 10% fetal bovine serum and 1% penicillin-streptomycin at 37 °C in atmosphere containing 5% CO_2_. Cells were passed every 3 days at a density of 10^6^ cells per 10-cm dish following the 3 T3 protocol. All experiments were performed on spontaneously immortalized primary MEFs post-senescence after passage 20 at 50-70% confluence. For starvation experiments MEFs were incubated for the indicated time in EBSS. Rapamycin and chloroquine diphosphate (CQ) were used to inhibit mTOR and autophagy respectively. EBSS (E2888), chloroquine diphosphate and rapamycin were purchased from (Sigma Aldrich St. Louis, MO).

### siRNA Transfection

*Klf4*^+/+^ MEFs were grown to 60% confluency in a 6-well plate before siRNA transfection. siRNA against *Klf4* was obtained as Silencer® Select siRNA against *Klf4* (Life Technologies, Grand Island, NY, USA) and dissolved to a stock solution of 50 μM with water. MEFs were transfected with up to 10 nM siRNA against *Klf4* or negative control siRNA provided by the manufacturer using Lipofectamine 3000 (Life Technologies, Grand Island, NY, USA) as the transfection reagent following the manufacturer’s protocol after 24 hrs for collection for analysis.

### Plasmid Transfection

To overexpress *Klf4*, MEFs were transiently transfected with 4 μg DNA plasmids containing Klf4-GFP or GFP as a control in 6-well plate. Lipofectamine 3000 reagent (Life Technologies, Grand Island, NY, USA) was used following manufacturer’s protocol. Cells were photographed and collected for immunoblotting after indicated time. Transfection efficiency was examined under an Olympus IX51 microscope for GFP fluorescence 24 hrs post transfection. To study the role of KLF4 in autophagy, we transiently transfected *Klf4*^+/+^ and *Klf4*^−/−^ MEFs with EGFP-LC3 plasmids and cells were treated with either DMSO or rapamycin for 6 hrs. The percentage of cells with punctate pattern of EGFP-LC3B was counted by fluorescence microscopy. The EGFP-LC3 was obtained from Addgene, Incorporated (plasmid 11546) [48] and constructed in the laboratory of K. Kirkegaard, Stanford University. Transfection efficiency was about 40% by visualizing GFP signal under fluorescent microscope. Transfection resulted in an overexpression of KLF4 in both *Klf4*^+/+^ and *Klf4*^*−/−*^ MEFs as indicated by Western blot (Additional file 2 Figure S2). The overexpression of *Klf4* was comparable to a previous report [14].

### Western blot analysis

Cell protein extraction and Western blot analyses were performed using standard procedures. Briefly, protein samples were prepared by lysing cells with 100 mM Tris–HCl, pH 6.8, 2% SDS, 100 mM dithiothreitol, 0.01% bromphenol blue, and 10% glycerol and heating to 98 °C for 5 min to denature the proteins. The proteins were loaded into Mini-PROTEAN® TGX™ Precast Gels (Bio-Rad Laboratories, Hercules, CA) in Tri/Glycine/SDS buffer (Bio-Rad Laboratories, Hercules, CA). After electrophoresis, the SDS-polyacrylamide gel was transferred to a nitrocellulose membrane in Tris/glycine transfer buffer (Bio-Rad Laboratories, Hercules, CA) with 10% methanol. The membranes were immunoblotted with the following primary antibodies against: LC3, KLF4, β-Actin (Abcam, Cambridge, MA, USA), p21 (BD Pharmingen, Franklin Lakes, NJ, USA), and phospho-p70S6K and γ-H2AX (Cell Signaling, Danvers, MA, USA). The blots were then incubated with the appropriate horseradish peroxidase-conjugated secondary antibodies for 1 h at room temperature. Anti-rabbit secondary antibody and anti-mouse secondary antibody were purchased from Cell Signaling (Danvers, MA, USA) and Abcam (Cambridge, MA, USA), respectively. The antibody-antigen complex was visualized by an Immun-Star™ HRP Chemiluminescence Kit and ChemiDoc™ XRS+ System (Bio-Rad Laboratories, Hercules, CA). The intensities of the bands were quantified using volume tools in Gel Imager program by normalizing the band intensity of protein of interest to that of β-Actin.

### Trypan Blue Assay

MEFs with indicated treatments were trypsinized and resuspended in PBS. Resuspended MEFs were mixed with 0.4% Trypan Blue solution (Sigma Aldrich St. Louis, MO) and let stain for 5 min at room temperature. The number of stained MEFs was then quantified with a hemocytometer under bright field under a microscope.

### Flow Cytometry

Flow cytometry to quantify apoptotic cell population was performed as previously described [10]. Briefly, *Klf4*^+/+^ and *Klf4*^−/−^ MEFs were plated in 6-well plates at 10^5^ cells/well. The next day cells were treated with full media, 10 μM CQ, EBSS or both 10 μM CQ and EBSS. After 8 hours treatment, cells were rinsed in PBS, trypsinized, and pelleted. Cells were fixed in 70% ethanol in PBS and incubated −20 °C overnight. The fixed cells were pelleted and resuspended in PBS that contain 50 mg/ml propidium iodide, 50 mg/ml RNase A, 0.1% Triton X-100 and 0.1 mM ethylene diaminetetraacetic acid at room temperature for 30 min before analysis. Cell-cycle profile analysis was performed on analyzed with a BD Accuri™ D Biosciences C6 Flow Cytometer (San Jose, CA, USA). Cellular debris was gated out of the analysis using forward and side scatter (Additional file 3 Figure S3). Apoptotic cells were identified by their sub-G1 DNA content.

### Immunostaining

Immunostaining was carried out as previously described with modification [14]. MEFs grown on chamber slides were washed briefly with PBS. They were then fixed with 3.7% methanol-free formaldehyde for 30 min at room temperature. MEFs were washed three times with PBS and incubated with blocking solution (3% bovine serum albumin (BSA), 0.2% Triton X-100 in PBS) for 1 h at room temperature. Rabbit anti-γ-H2AX primary antibody (Cell Signaling) diluted in blocking solution was incubated at room temperature for an hour. Primary antibody was detected with Alexa Fluor 488- conjugated goat anti-rabbit IgG antibody (Santa Cruz) for 1 h at room temperature. Cells were then washed once and counterstained with DAPI (Life Technologies) for 5 min at room temperature in the dark. Finally cells were washed three times, mounted in Prolong Antifade kit (Molecular Probes), and visualized with a Zeiss 710 confocal laser scanning microscope (Carl Zeiss, Thornwood, NY). The validity of the immunostaining signal was confirmed by the negative control, which was not incubated in primary antibody, and the positive control, which was treated with etoposide to induce DNA damage (Additional file 4 Figure S4).

### Quantitative real-time PCR analysis

Total RNA from cultured *Klf4*^+/+^ and *Klf4*^−/−^ MEFs was isolated using RNeasy1 Mini Kit (Qiagen, Valencia, CA) according to the manufacturer’s protocol. RNA was subjected to gDNA elimination columns (Qiagen, Valencia, CA) in order to remove any contaminating genomic DNA. cDNA was prepared from 500 ng of RNA and amplified with Omniscript RT Kit (Qiagen) and polyT primer (Integrated DNA Technologies, Coralville, IA). Synthesized cDNA was subjected for RT-qPCR analysis using SYBR® Green PCR Master Mix for 40 cycles (Life Technologies) following manufacturer’s protocol. The expression of *Cdkn1a* or *Atg7* was normalized to the expression level of *β-Actin*. Relative fold change in gene expression level was calculated by comparing the normalized gene expression in *Klf4*^−/−^ MEFs to that in *Klf4*^+/+^ MEFs, or comparing the Klf4-transfected MEFs to GFP-transfected MEFs. The gene expression levels of *Klf4*^+/+^ or GFP-transfected MEFs were set to 1. Data shown represents two independent experiments, each performed in triplicates. PCR reactions were performed using the following primers purchased from Integrated DNA Technologies (Coralville, IA). *Atg7* F: 5’ TCT GGG AAG CCA TAA AGT CAG G 3’; *Atg7* R: 5’ GCG AAG GTC AGG AGA A 3’; *Cdkn1a* F: 5’ ATC ACC AGG ATT GGA CAT GG 3’; *Cdkn1a* R: 5’ CGG TGT CAG AGT CTA GGG GA 3’; *β-Actin* F: 5’ ATG GAG GGG AAT ACA GCC C 3’; *β-Actin* R: 5’ TTC TTT GCA GCT CCT TCG TT 3’.

### Statistics

All experiments were done independently at least three times unless otherwise indicated. Student’s t-test was used to analyze statistical significance. P-values lower than 0.05 were considered significant.

## Additional files

Additional file 1: Figure S1. Morphology and relative number of cells in MEFs. *Klf4* ^*+/+*^ *and Klf4* ^*−/−*^ MEFs incubated in full-media (FM) were allowed to seed for 24 hours. Cells then were photographed using Olympus IX51 microscope at 2, 4, 8, 12, and 24 hours. Showing is relative numbers of cells under different confluency. Additional file 2: Figure S2. Klf4-GFP plasmid overexpression in MEFs. Transfection resulted in an overexpression of KLF4 in MEFs as indicated by western blot **(A)** wild-type **(B)** *Klf4*-null. We compared the levels of KLF4 in *Klf4* ^*+/+*^ and *Klf4* ^*−/−*^ MEFs that transfected with either Klf4-GFP or GFP as a control. The expression of KLF4 is highly increased in both *Klf4* ^*+/+*^ and *Klf4* ^*−/−*^ MEFs that transfected with Klf4-GFP as compared to GFP-control-transfected *Klf4* ^*+/+*^ and *Klf4* ^*−/−*^ MEFs (Lane A1 and Lane B3). Endogenous levels of Klf4 showed at 55 KD where as Klf4 conjugated with GFP showed at 82 KD **(A)**. Materials and Methods Plasmid were constructed as previously described in [14]. Additional file 3: Figure S3. Flow cytometry analysis of MEFs. Flow cytometry studies were performed in MEFs as described in Materials and Methods. Briefly, *Klf4* ^+/+^ and *Klf4* ^−/−^ MEFs were plated in 6-well plates at 10^5^ cells/well. The next day cells were treated with full media, 10 μM CQ, EBSS or both 10 μM CQ and EBSS. *Cellular debris was gated out of the analysis using forward and side scatter to prevent against cellular debris from interfering with the quality of our results. Klf4* ^+/+^ ***(A - D)*** *and Klf4* ^−/−^ ***(E - H)*** *.*
Additional file 4: Figure S4. Control for immunofluorescence labeling. Immunofluorescence studies were performed in MEFs as described in Materials and Methods. Cells were counterstained with DAPI to visualize the nuclei. MEFs were exposed to etoposide for 5 hours and collected for γH2AX **(A)** immunostaining and **(B)** western blot. MEFs treated with etoposide for 5hrs to acquired greater levels of DNA damage as compared with DMSO treated control MEFs as indicated by γH2AX (lanes 1 and 2). An increase in γH2AX immunostaining was also observed as positive control. The validity of the immunostaining signal was confirmed by the negative control, which was not incubated in primary antibody.

## Abbreviations

BSA: Bovine serum albumin
CQ: Chloroquine Diphosphate
DAPI: 4^’^,6-Diamidino-2-phenylindol
DMEM: Dulbecco’s modified Eagle’s Medium
DMSO: Dimethyl sulfoxide
EBSS: Earle’s Balanced Salt Solution
FBS: Fetal bovine serum
GFP: Green fluorescent protein
KLF4: Krüppel-like factor 4
LC3: Light chain 3
MEFs: Mouse embryonic fibroblasts
mTOR: Mammalian target of rapamycin
PBS: Phosphate buffered saline
ROS: Reactive oxygen species
ATG: Autophagy-related genes.
Cdkn1a: Cyclin-dependent kinase inhibitor 1A
