# Supplementary figures and images for: Impaired autophagy in mouse embryonic fibroblasts null for Krüppel-like Factor 4 promotes DNA damage and increases apoptosis upon serum starvation
Source: Mol Cancer. 2015 May 6;14:101. doi: 10.1186/s12943-015-0373-6 (PMC4422415; doi:10.1186/s12943-015-0373-6)

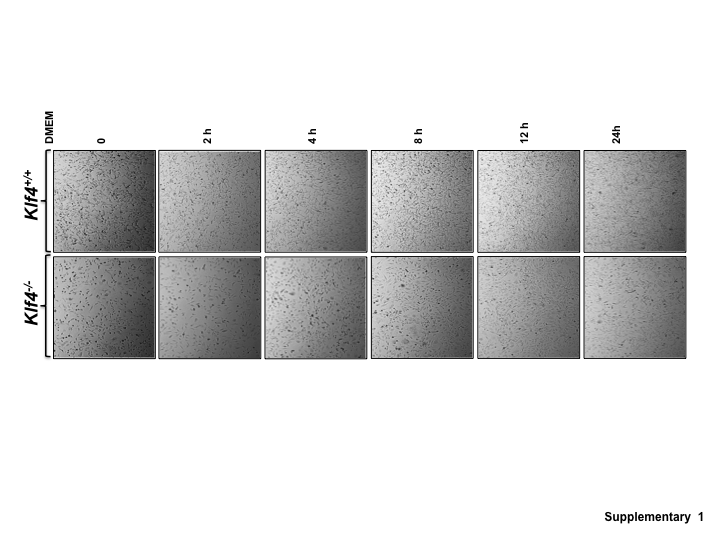

Supplement: Additional file 1: Figure S1. — Morphology and relative number of cells in MEFs. Klf4 +/+ and Klf4 −/− MEFs incubated in full-media (FM) were allowed to seed for 24 hours. Cells then were photographed using Olympus IX51 microscope at 2, 4, 8, 12, and 24 hours. Showing is relative numbers of cells under different confluency. [file 12943_2015_373_MOESM1_ESM.tiff]

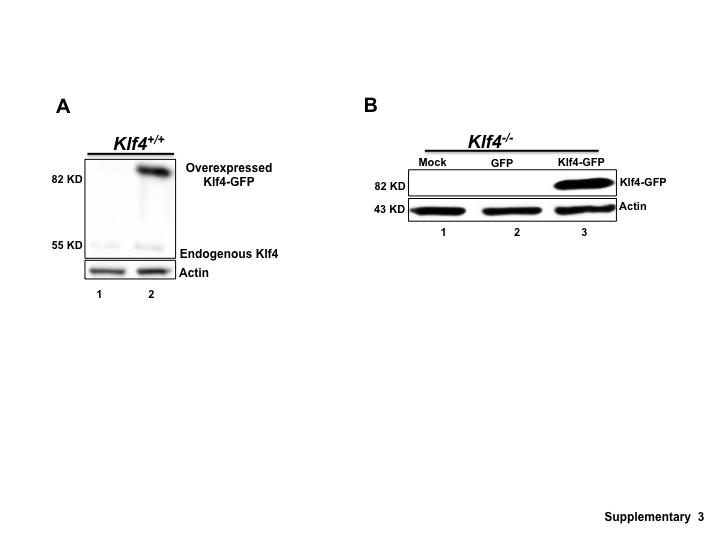

Supplement: Additional file 2: Figure S2. — Klf4-GFP plasmid overexpression in MEFs. Transfection resulted in an overexpression of KLF4 in MEFs as indicated by western blot (A) wild-type (B) Klf4-null. We compared the levels of KLF4 in Klf4 +/+ and Klf4 −/− MEFs that transfected with either Klf4-GFP or GFP as a control. The expression of KLF4 is highly increased in both Klf4 +/+ and Klf4 −/− MEFs that transfected with Klf4-GFP as compared to GFP-control-transfected Klf4 +/+ and Klf4 −/− MEFs (Lane A1 and Lane B3). Endogenous levels of Klf4 showed at 55 KD where as Klf4 conjugated with GFP showed at 82 KD (A). Materials and Methods Plasmid were constructed as previously described in [14]. [file 12943_2015_373_MOESM2_ESM.tiff]

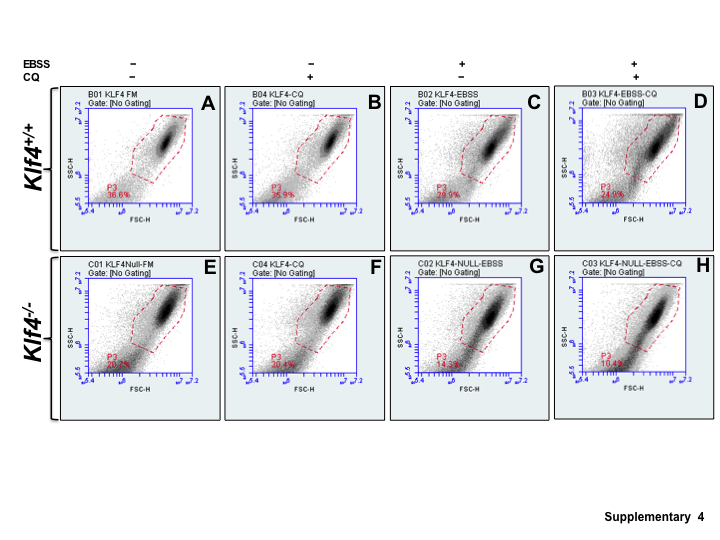

Supplement: Additional file 3: Figure S3. — Flow cytometry analysis of MEFs. Flow cytometry studies were performed in MEFs as described in Materials and Methods. Briefly, Klf4 +/+ and Klf4 −/− MEFs were plated in 6-well plates at 105 cells/well. The next day cells were treated with full media, 10 μM CQ, EBSS or both 10 μM CQ and EBSS. Cellular debris was gated out of the analysis using forward and side scatter to prevent against cellular debris from interfering with the quality of our results. Klf4 +/+ (A - D) and Klf4 −/− (E - H) . [file 12943_2015_373_MOESM3_ESM.tiff]

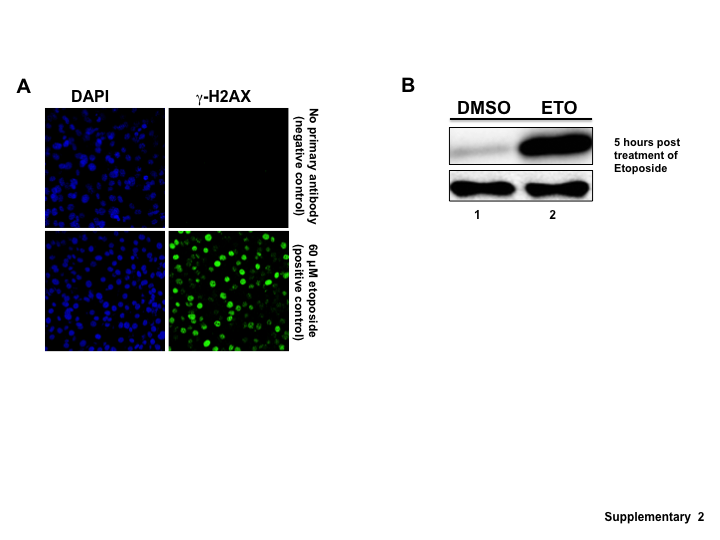

Supplement: Additional file 4: Figure S4. — Control for immunofluorescence labeling. Immunofluorescence studies were performed in MEFs as described in Materials and Methods. Cells were counterstained with DAPI to visualize the nuclei. MEFs were exposed to etoposide for 5 hours and collected for γH2AX (A) immunostaining and (B) western blot. MEFs treated with etoposide for 5hrs to acquired greater levels of DNA damage as compared with DMSO treated control MEFs as indicated by γH2AX (lanes 1 and 2). An increase in γH2AX immunostaining was also observed as positive control. The validity of the immunostaining signal was confirmed by the negative control, which was not incubated in primary antibody. [file 12943_2015_373_MOESM4_ESM.tiff]
